# Brainstem projections of neurons located in various subdivisions of the dorsolateral hypothalamic area—an anterograde tract-tracing study

**DOI:** 10.3389/fnana.2014.00034

**Published:** 2014-05-16

**Authors:** Rege S. Papp, Miklós Palkovits

**Affiliations:** ^1^Neuromorphological and Neuroendocrine Research Laboratory, Department of Anatomy, Histology and Embryology, Semmelweis University and the Hungarian Academy of SciencesBudapest, Hungary; ^2^Human Brain Tissue Bank and Laboratory, Semmelweis UniversityBudapest, Hungary

**Keywords:** dorsolateral hypothalamus, anterograde tract-tracing, BDA, rat, lower brainstem

## Abstract

The projections from the dorsolateral hypothalamic area (DLH) to the lower brainstem have been investigated by using biotinylated dextran amine (BDA), an anterograde tracer in rats. The DLH can be divided into 3 areas (dorsomedial hypothalamus, perifornical area, lateral hypothalamic area), and further subdivided into 8 subdivisions. After unilateral stereotaxic injections of BDA into individual DLH subdivisions, the correct sites of injections were controlled histologically, and the distribution patterns of BDA-positive fibers were mapped on serial sections between the hypothalamus and spinal cord in 22 rats. BDA-labeled fibers were observable over 100 different brainstem areas, nuclei, or subdivisions. Injections into the 8 DLH subdivisions established distinct topographical patterns. In general, the density of labeled fibers was low in the lower brainstem. High density of fibers was seen only 4 of the 116 areas: in the lateral and ventrolateral parts of the periaqueductal gray, the Barrington's, and the pedunculopontine tegmental nuclei. All of the biogenic amine cell groups in the lower brainstem (9 noradrenaline, 3 adrenaline, and 9 serotonin cell groups) received labeled fibers, some of them from all, or at least 7 DLH subdivisions, mainly from perifornical and ventral lateral hypothalamic neurons. Some of the tegmental nuclei and nuclei of the reticular formation were widely innervated, although the density of the BDA-labeled fibers was generally low. No definitive descending BDA-positive pathway, but long-run solitaire BDA-labeled fibers were seen in the lower brainstem. These descending fibers joined some of the large tracts or fasciculi in the brainstem. The distribution pattern of BDA-positive fibers of DLH origin throughout the lower brainstem was comparable to patterns of previously published orexin- or melanin-concentrating hormone-immunoreactive fibers with somewhat differences.

## Introduction

The dorsolateral hypothalamic area (DLH) covers a relatively large, but not strictly outlined area in the posterior part of the hypothalamus. This area extends rostrocaudally from the level of the caudal aspect of the paraventricular nucleus (about 2.4 mm caudal to the bregma level, in adult rats) until the level of the caudal end the third ventricle (3.4 mm caudal to the bregma).

**Figure 1:**
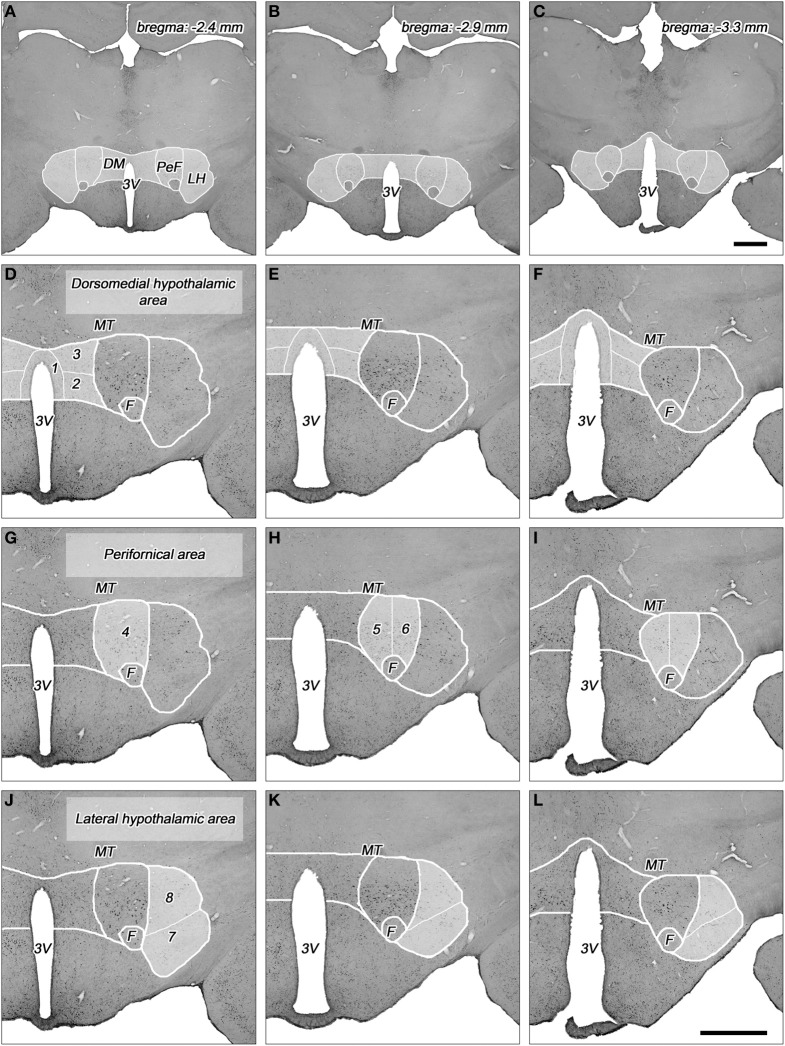
**Topography of the areas and their subdivisions in the dorsolateral hypothalamus. (A–C)** Cross-sectional profile of the dorsolateral hypothalamus (DLH) in three coronal sections at various levels caudal to the bregma (indicated on the right top). Higher magnifications of DLH of **(A)**, **(B)**, and **(C)** are shown in panels **(D,G,J)**, **(E,H,K)**, and **(F,I,L)**, respectively. **(D–L)** The subdivisions of the dorsomedial hypothalamic **(D–F)**, perifornical **(G–I)**, and lateral hypothalamic areas **(J–L)** are numbered and indicated with white lines in faint white background. (1) Periventricular area, (2) dorsomedial nucleus, (3) dorsomedial hypothalamus, (4) rostral perifornical area, (5) caudomedial perifornical area, (6) caudolateral perifornical area, (7) ventral part of the lateral hypothalamus, (8) dorsal part of the lateral hypothalamus. 3V, third ventricle; DM, dorsomedial hypothalamic area; F, fornix; LH, lateral hypothalamic area; MT, mamillothalamic tract; PeF, perifornical area. Scale bars: 1 mm.

The mediolateral and dorsoventral extension of the DLH is a subject of debate since no exact topographical borderlines exist. The area reaches the midline and slightly over the third ventricle, including the top portion of the posterior periventricular nucleus. From here, the borderline arches over the major portion of the dorsomedial nucleus and along the lateral border of the ventromedial nucleus it reaches the ventral surface of the hypothalamus. The dorsolateral borderline may be traceable just under the thalamus and the zona incerta, as lateral as the medial edge of the internal capsule/cerebral peduncle (Figure 1). The DLH incorporates a part of the posterior periventricular zone (also called nucleus), an area between the dorsomedial and perifornical nuclei, the extended perifornical area (PeF; that is larger than the perifornical nucleus itself), and a substantial (posterior) portion of the lateral hypothalamic area. In some descriptions, the entire dorsal part of the dorsomedial nucleus is also included to the DLH since the orexin- and melanin-concentrating hormone (MCH)-expressing neurons, the two major neuropeptides that spread over the entire DLH do not respect the borderlines of the dorsal part of the dorsomedial nucleus, and also extended into the medial part of the zona incerta and the ventral portion of the posterior hypothalamic nucleus (Skofitsch et al., 1985; Bittencourt et al., 1992; Peyron et al., 1998; Nambu et al., 1999).

**Figure 2:**
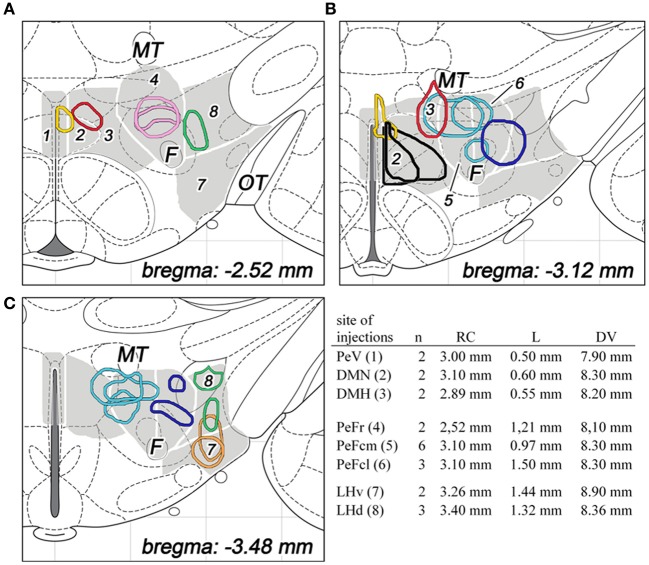
**The sites and extensions of BDA injections in various parts of the dorsolateral hypothalamus are shown on coronal sections of the hypothalamus, based on drawings from the atlas of Paxinos and Watson (2007)**. The different regions of the dorsolateral hypothalamus are numbered and indicated in gray background with white line borders in three different rostrocaudal levels **(A–C)**. The injection sites are colored as follow: periventricular area (PeV, yellow), rostral perifornical area (PeFr, pink), caudomedial perifornical area (PeFcm, light blue), caudolateral perifornical area (PeFcl, dark blue), dorsomedial nucleus (DMN, black), dorsomedial hypothalamus (DMH, red), ventral part of the lateral hypothalamus (LHv, orange), and dorsal part of the lateral hypothalamus (LHd, green). Distances from the level of the bregma are indicated on bottom right. The number of animals and stereotaxic coordinates of tracer injections are given on the right. Rostrocaudal coordinates (RC) refer to distances caudal to the level of the bregma, lateral coordinates (L) refer distances lateral to the midline, while dorsoventral coordinates (DV) refer to ventral distances from the dura mater on the base. F, fornix; MT, mamillothalamic tract; OT, optic tract.

The DLH represents a heterogeneous area with subdivisions that are characterized on the basis of different neuronal cell types, different density of cell populations, and different neuronal in- and outputs. The DLH has been divided into two (Fadel et al., 2002; Baldo et al., 2004; Sunanaga et al., 2009; Zhang et al., 2009), three (Murphy et al., 2003; Nixon and Smale, 2004; Harris et al., 2005), four (Satoh et al., 2003), or even 13 (Swanson et al., 2005; Hahn, 2010) subdivisions. Based on our previous quantitative histological studies by measuring cell density on coronal serial sections of the hypothalamus in rat (Palkovits, 1975), and on quantitative topographical analysis of orexin (Papp et al., in preparation) and MCH-expressing neurons (Vas et al., in preparation) in the DLH, we divided the DLH into three areas with a total of 8 subdivisions (Table 1, Figures 1, 2). The *dorsomedial hypothalamic area* incorporates the upper portion of the posterior periventricular area (PeV), the lateral portion of the dorsal part of the dorsomedial nucleus (DMN), and the area between the dorsomedial and perifornical area, called dorsomedial hypothalamic subdivision (DMH). The *perifornical area* includes the rostral (PeFr), the caudomedial (PeFcm), and the caudolateral (PeFcl) subdivisions of the perifornical nucleus. The *lateral hypothalamic area* consists of two subdivisions in the posterior hypothalamus, one ventral (LHv) and one dorsal (LHd) lateral hypothalamic subdivisions separated by a virtual line between ventral edge of the fornix and the medial edge of the pedunculus cerebri/capsula interna (Figure 1). The detailed description of the topography of the subdivisions are in a separate subchapter in the “Discussion” and in the Figure 1. The parceling of the DLH is somewhat subjective, but one can easily identify the borderlines of the subdivisions.

**Table 1:** **Relative density of BDA-labeled fibers in the lower brainstem after tracer injections into different subregions of the dorsolateral hypothalamus**.

	**Dorsomedial hypothalamic area**			**Perifornical area**			**Lateral hypothalamic area**	
	**PeV**	**DMN**	**DMH**	**PeFr**	**PeFcm**	**PeFcl**	**LHv**	**LHd**
**PERIAQUEDUCTAL GRAY**								
Periaqueductal gray, ventrolateral part	++	++	++	+++	+++	++	++	+
Periaqueductal gray, lateral part	+++	+	++	+++	+++	+++	++	+
Periaqueductal gray, dorsomedial part	++	+	+	±	+	±	−	±
Periaqueductal gray, dorsolateral part	+	+	+	+	++	−	±	+
**SOMATO- AND VISCEROMOTOR NUCLEI**								
Oculomotor nucleus	±	−	+	±	±	−	−	−
Trochlear nucleus	±	−	±	±	+	±	+	±
Paratrochlear nucleus	−	−	±	±	+	+	+	−
Abducens nucleus	−	−	−	−	±	±	−	±
Precuneiform area	+	−	+	+	++	+	−	−
Motor trigeminal nucleus	−	−	−	±	±	±	−	±
Motor facial nucleus	−	−	−	−	−	±	−	−
Perifacial zone	−	−	−	±	+	+	+	+
Ambiguus nucleus	±	−	±	±	−	±	±	±
Retroambiguus nucleus	−	−	±	−	±	±	+	−
Hypoglossal nucleus	−	−	−	±	±	±	+	±
Edinger-Westphal nucleus	+	±	±	+	+	+	+	+
Superior salivatory nucleus	±	−	+	+	+	±	+	+
Inferior salivatory nucleus	−	−	−	+	−	±	±	−
Dorsal motor nucleus of vagus	−	+	−	+	+	±	+	+
**SOMATO- AND VISCEROSENSORY NUCLEI**								
Spinal trigeminal nucleus	−	−	−	−	−	±	±	−
Supratrigeminal nucleus	−	−	−	+	+	+	+	±
Peritrigeminal zone	−	−	±	±	±	±	±	−
Nucleus of the solitary tract	+	±	−	+	±	±	+	±
Trigeminal-solitary transition zone	−	−	−	+	±	±	−	+
Sagulum nucleus	−	−	−	−	−	±	−	−
Juxtaolivary nucleus	−	−	−	−	+	±	±	−
Superior olive (lateral)	−	−	−	+	−	−	−	−
Caudal periolivary nucleus	−	−	−	−	±	−	−	−
Nucleus of the trapezoid body	−	−	−	−	±	−	−	−
Nuclei of the lateral lemniscus	−	−	−	−	−	−	+	−
Perilemniscal nucleus, ventral part	−	−	−	−	±	±	−	±
Medial vestibular nucl., magnocell. part	−	−	−	+	±	±	−	−
Supragenual nucleus	±	−	−	±	−	−	±	−
**MONOAMINE CELL GROUPS**								
Dorsal raphe nucleus	+	+	+	++	++	++	++	+
Median raphe nucleus	±	+	±	±	+	+	±	−
Rhabdoid nucleus	−	−	±	−	±	±	−	+
Pontine raphe nucleus	−	−	±	−	+	+	+	±
Raphe magnus nucleus	+	+	+	++	++	++	++	±
Raphe pallidus nucleus	++	+	−	+	++	++	+	+
Parapyramidal nucleus	−	−	−	±	±	±	±	±
Raphe obscurus nucleus	+	−	±	+	+	+	±	±
Paramedian raphe nucleus	+	±	±	±	+	+	+	±
A1 noradrenaline cell group, rostral part	++	−	−	+	±	+	++	−
A1 noradrenaline cell group, caudal part	+	+	±	+	+	+	+	−
A2 noradrenaline cell group	+	+	±	+	+	+	±	±
A5 noradrenaline cell group	−	−	+	+	+	+	+	±
Locus coeruleus	++	+	±	++	++	++	+	+
Subcoeruleus area, dorsal part	+	−	±	++	++	+	++	±
Subcoeruleus area, ventral part	−	−	±	+	+	+	±	±
Subcoeruleus area, alpha part	±	−	±	+	+	+	+	+
A7 noradrenaline cell group	−	−	+	±	±	+	±	−
C1 adrenaline cell group	−	+	±	++	±	+	+	+
C2 adrenaline cell group	−	−	−	±	+	±	±	±
C3 adrenaline cell group	−	−	−	+	±	−	+	−
**PONS EXCEPT RETICULAR FORMATION**								
Deep mesencephalic nucleus	+	−	±	−	−	±	±	−
Medial paralemniscal nucleus	−	−	±	−	±	±	±	−
Pontine nuclei	−	−	−	±	±	±	−	−
Barrington's nucleus	+++	+	+	+	+	+	±	−
Lateral parabrachial nucleus	+	±	±	++	+	++	+	±
Medial parabrachial nucleus	+	+	±	++	+	++	+	+
Kölliker-Fuse nucleus	−	−	+	±	±	+	+	−
Ventral tegmental nucleus	+	−	±	±	+	+	+	+
Dorsal tegmental nucleus, central part	−	−	−	−	±	−	−	−
Dorsal tegmental nucl., pericentral part	−	−	−	±	+	±	−	±
Dorsal pedunculopontine nucleus	−	−	−	+	+	−	−	−
Laterodorsal tegmental nucleus	+	+	+	+	++	++	++	±
Pedunculopontine tegmental nucleus	+	−	+	+	+	++	+++	−
Dorsomedial tegmental area	+	−	±	±	+	+	±	+
Subpeduncular tegmental nucleus	+	±	±	±	+	±	++	−
Sphenoid nucleus	−	−	−	−	±	±	−	+
Interstit. nucl. of the med. longitud. fasc.	−	−	−	−	−	±	±	−
**MEDULLA EXCEPT RETICULAR FORMATION**								
Nucleus X	−	−	−	−	−	−	−	±
Inferior olive	−	−	−	±	−	±	−	−
Epifascicular nucleus	−	−	−	+	±	±	−	−
Prepositus nucleus	−	−	−	±	±	±	±	±
Bötzinger complex	−	−	±	±	−	±	+	−
Pre-Bötzinger complex	−	−	±	±	±	+	++	±
Rostral ventral respiratory group	−	−	±	±	−	+	+	+
Nucleus of Roller	−	−	−	±	±	−	−	−
Linear nucleus of the medulla oblongata	−	−	−	±	−	±	−	−
Parasolitary nucleus	−	−	−	−	−	±	−	−
Central cervical nucl. of the spinal cord	−	−	−	±	±	±	−	±
**RETICULAR FORMATION**								
Isthmic reticular formation	+	−	+	++	++	+	++	+
Pontine reticular nucleus, oral part	+	−	±	+	++	++	+	+
Pontine reticular nucleus, caudal part	+	±	±	+	+	++	+	−
Pontine reticular nucleus, ventral part	+	−	±	+	+	+	+	±
Paramedian pontine reticular formation	−	−	±	−	−	−	±	±
Parvicellular reticular nucleus	±	±	+	++	−	−	−	+
Parvicellular reticular nucleus, alpha part	+	−	±	+	+	±	−	−
Reticulotegmental nucleus of the pons	±	−	−	±	+	+	−	±
Intermediate reticular nucleus	+	−	+	++	+	+	++	+
Dorsal paragigantocellular nucleus	−	−	−	±	±	+	±	±
Lateral paragigantocellular nucleus	±	−	±	±	+	++	++	++
Lateral paragigantocell. nucl., external part	+	±	−	±	+	±	−	+
Lateral paragigantocell. nucl., alpha part	−	±	±	±	+	+	+	−
Gigantocellular reticular nucleus	−	−	+	+	+	+	+	+
Gigantocellular ret. nucleus, alpha part	±	−	±	+	+	+	++	±
Gigantocellular ret. nucleus, ventral part	+	−	−	++	+	+	±	+
Paramedian reticular nucleus	−	−	±	−	±	±	−	−
Lateral reticular nucleus	∓	−	±	±	±	±	+	±
Medullary reticular nucleus, dorsal part	+	−	+	+	+	+	+	±
Medullary reticular nucleus, ventral part	−	−	−	+	±	±	+	±
**BDA FIBERS IN NEURONAL TRACTS OR FASCICULI**								
Medial lemniscus	++	+	+	±	+	++	+	±
Trigeminothalamic tract	−	−	±	+	+	±	±	−
Mesencephalic trigeminal tract	−	−	−	±	−	−	±	±
Medial longitudinal fasciculus (midbrain)	−	−	±	+	++	++	+	±
Medial longitudinal fasciculus (pons)	−	−	−	±	±	±	−	−
Vestibulomesencephalic tract	−	−	−	+	±	±	±	±
Vestibulospinal tract	−	−	−	±	±	±	−	−
Tectospinal tract	−	−	±	±	±	±	±	+
Rubrospinal tract	+	−	−	+	+	±	±	−
Ventral spinocerebellar tract	−	−	±	±	−	−	−	−
Facial nerve (intracranial part)	−	−	−	±	−	−	−	−
Superior cerebellar peduncle	+	±	+	++	++	+	+	±
Pyramidal tract	−	−	−	±	−	±	−	+
Solitary tract	−	−	−	±	−	±	±	±

DMH, dorsomedial hypothalamus; DMN, dorsomedial nucleus; LHd, dorsal part of the lateral hypothalamic area; LHv, ventral part of the lateral hypothalamic area; PeFcl, lateral part of the caudal perifornical area; PeFcm, medial part of the caudal perifornical area; PeFr, rostral perifornical area; PeV, periventricular area. (Nomenclature in the table is partly based on names used by Paxinos and Watson, 2007.)

Neuronal projections from the DLH to the lower brainstem have been visualized by various types of antero- and retrograde tract-tracing studies, in detail (Veening et al., 1987; Elias et al., 1998; Goto et al., 2005; Hahn and Swanson, 2010, 2012). The projections of neurons from the *discrete subdivisions* of the DLH have not been investigated *individually*. The question was raised whether neurons in the distinct DLH subdivisions have specific projection patterns in the lower brainstem that would indicate specific functional role of these neurons? To reveal the answer, biotinylated dextran amine (BDA), an anterograde tracer was stereotaxically injected into the 8 subdivisions of the DLH unilaterally in 8 different groups of rats, and projections into the distinct areas and nuclei of the lower brainstem have been mapped, systematically. For the evaluation of the distribution of BDA-labeled fibers on serial sections, the atlas of Paxinos and Watson (2007) has been used. Among the 194 areas/nuclei/subdivisions showed up in the map, we found 116 that contained BDA-labeled fibers (Table 1).

## Materials and methods

### Animal housing

Adult male Wistar rats (250–300 g; Charles Rivers Laboratory, Isaszeg, Hungary) were used. All efforts were made to minimize the number of animals used, and also their suffering. Experiments were performed according to the European Communities Council Directive of 24 November 1986 (86/609/EEC) and the National Institutes of Health “Principles of Laboratory Animal Care” (NIH Publications No. 85-23, revised 1985), as well as specific national laws (the Hungarian Governmental Regulations on Animal Studies, December 31, 1998). Permission was obtained from the local Ethical Committee of the Semmelweis University, Budapest. Animals were housed three per cage, at a temperature of 22 ± 1°C, with 12 h light and 12 h dark periods (light from 6.00 AM) and have access to food and water *ad libitum*.

### Anterograde tract-tracing

For the surgical procedure, animals were anesthetized with a mixture of 1 ml/kg body weight ketamine (100 mg/ml; CP-Pharma, Burgdorf, Germany) and 0.6 ml/kg body weight xylazin (20 mg/ml; CP-Pharma), delivered intramuscularly. Under anesthesia, rats received a single stereotaxically placed iontophoretic injection of 10% BDA (10,000 MW; Molecular Probes, Eugene, OR, USA) prepared in 0.1 M phosphate buffer (PB), pH 7.4, in one of the 8 subdivisions of the DLH unilaterally. Glass capillaries were used with 18–22 μm tip diameter for the electrophoresis by applying 7 mA positive current pulses (7 s on, 7 s off) for 20 min; the capillaries were then left in place for 10 min before retraction. The number of animals and coordinates used for tracer injections are summarized in Figure 2.

### Tissue preparation and analysis

#### Perfusion, fixation

The animals were killed 13 days after surgical interventions. After anesthesia with a mixture of 1 ml/kg body weight ketamine (100 mg/ml; CP-Pharma) and 0.6 ml/kg body weight xylazin (20 mg/ml; CP-Pharma) delivered intramuscularly, rats were perfused transcardially with 0.9% saline followed by 300 ml of 4% paraformaldehyde, pH 7.4. The brains were removed, post-fixed overnight in a fresh solution, then cryoprotected with 20% sucrose for 24 h, at 4°C. Fifty μ m thick serial coronal sections were cut from the hypothalamus to the spinal cord on a frigomobile (Frigomobil, Reichert-Jung, Vienna, Austria), and the sections were used for immunohistochemistry and immunofluorescence visualizations of BDA-labeling.

#### Visualization of BDA

Every fourth section was stained for BDA using either immunoperoxidase or immunofluorescence procedures with or without tyramide-mediated amplification (Adams, 1992; Reiner et al., 2000). Free-floating sections were pretreated in 0.5% Triton X-100 for 1 h at room temperature. Sections were then incubated in avidin-biotin-horseradish peroxidase complex (ABC) at 1:500 dilution (Vectastain ABC Elite kit, Vector Laboratories, Burlingame, CA, USA) for 1 h. For biotin-tyramide amplification, sections were rinsed extensively first in PB and then in 0.05 M Tris buffer at pH 8.2 (TB), then sections were placed in biotin-tyramide solution (1:20,000) prepared in TB containing 0.0015% hydrogen peroxide for 15 min. Then a second ABC incubation was conducted at 1:500 for 30 min. Finally, the BDA-fibers were visualized using nickel enhanced 3,3-diaminobenzidine (Ni-DAB Sigma, St. Louis, MO, USA), a chromogen reaction resulting in a dark blue reaction product. Alternatively, after the ABC reaction FITC-tyramide amplification was performed (FITC-tyramide 1:10,000; 0.0015% H_2_O_2_ in TB for 10 min). Ni-DAB sections were mounted on gelatin coated slides (Intersan Kft., Budapest, Hungary) and coverslipped with DPX Mountant (Fluka, Switzerland). Fluorescent sections were mounted onto non-coated preclean slides (Intersan Kft.), and coverslipped with Aqua-Poly/Mount (Polysciences, Inc., Warrington, USA).

Sites of tracer deposition within the DLH were depicted on a series of computerized standard drawings of the rat brain from the atlas of Paxinos and Watson (2007) with the help of Adobe Photoshop CS 8.0.

#### Histological analysis of BDA labeling

Stained sections were examined with an Olympus BX60 microscope equipped with fluorescent epi-illumination. In a complete set of serial sections with 200 μm interval between the sections (every fourth 50 μm thick section), the density of the BDA-labeled fibers in the lower brainstem was assessed in each section. The relative abundance of BDA-labeled fibers was evaluated by the following grading: absence of labeled fibers (−), very low (±), low (+), moderate (++), and high (+++), see Table 1. The data from individually evaluated brains were grouped based on the given subdivision. The data were estimated in the following aspects: (1) which and how many of the brainstem areas/nuclei/subdivisions showed labeled fibers after injections into the 8 investigated DLH subdivisions; (2) the participation of the 8 DLH subdivisions in the innervations of the 116 brainstem areas/nuclei/subdivisions (how many subdivisions project to a particular brainstem subdivisions); (3) which brainstem areas/nuclei/subdivisions showed up high densities of labeled fibers; (4) evaluation of BDA fiber density in the special cell groups of the brainstem (innervations of biogenic amines, reticular formation, etc., see grouping in Table 1).

## Results

### Localization of the injection sites

A total number of 42 rats were BDA-injected. The number of animals *per* subdivisions, the stereotaxic coordinates and the extensions of the injected areas are summarized in Figure 2. Finally, 22 brains of 42 were considered acceptable for further cut (from the hypothalamus until the spinal cord) and mapping BDA projections in the lower brainstem. The correct sites and topography within the proper DLH subdivisions consisted the basic criteria for acceptance.

#### Dorsomedial hypothalamic area

Totally, six rats were found with topographically correct injection sites: two in each of the 3 subdivisions of the dorsomedial hypothalamic area.

**Figure 3:**
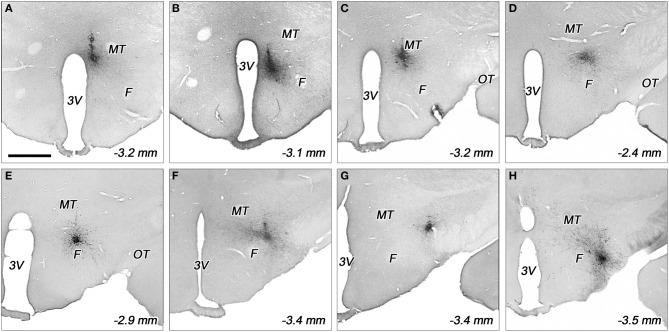
**Representative biotinylated dextran amine (BDA) injection sites in various subdivisions of the dorsolateral hypothalamus**. The injection sites located in the periventricular area **(A)**, dorsomedial nucleus **(B)**, dorsomedial hypothalamus **(C)**, rostral perifornical area **(D)**, caudomedial perifornical area **(E)**, caudolateral perifornical area **(F)**, dorsal **(G)**, and ventral parts of the lateral hypothalamus **(H)**. F, fornix; MT, mamillothalamic tract; OT, optic tract; 3V, third ventricle. Distances caudal to the bregma level are indicated on bottom right. Scale bar (for **A–H**): 1 mm.

In the case of *periventricular area*, the injected areas were relatively small, their average mediolateral diameters did not exceed 250 μm in the coronal plane, but they extended more in rostrocaudal and posteroventral directions. The injected area was located immediately adjacent to the third ventricle. They extended over the ventricle, dorsally. This division showed a small overlap with the dorsal part of the dorsomedial nucleus (Figures 2A,B, 3A).

Regarding to the *dorsal part of the dorsomedial nucleus*, the sizes of injections varied between 400 and 500 μm (Figures 2B, 3B). In one case, the injection spread in a relatively large area, covering almost the entire dorsomedial part of the dorsomedial nucleus. In the second rat, the injection's site was smaller. In both cases, the tracer spread slightly into the posterior periventricular area.

The sizes of the injections in *dorsomedial hypothalamic subdivision* were small, between 350 and 400 μm average diameters (Figures 2A,B, 3C). The injections centered in the dorsomedial hypothalamic area between the dorsomedial nucleus and the medial border of the perifornical area. BDA spread slightly into these two neighboring areas.

#### Perifornical area

Eleven injections were classified into the perifornical area (Figures 2A–C, 3D–F): two injections in the rostral (PeFr), six in the caudomedial (PeFcm), and three in the caudolateral (PeFcl) perifornical areas.

The sizes of injections in the *rostral perifornical area* were 350 and 450 μm. In adult rats, this area occupies a territory between the fornix and the most medial part of the zona incerta from the dorsomedial nucleus until the medial border of the lateral hypothalamic area (Figure 1G). Rostro-caudally, it extends between 2.4 and 2.8 mm caudal to the level of the bregma. In both cases, the injected BDA spread entirely within the boundaries of the area (Figures 2A, 3D).

A large area over the fornix with a rostro-caudal extension between 2.8 and 3.4 mm caudal to the bregma represents the caudal portion of the perifornical area (Figures 1H,I). An imaginary vertical line through the fornix divides this field into medial and lateral areas (Figures 1H,I, 2B,C). Regarding to the *caudomedial part of the perifornical area*, the average diameter of the injections varied between 250 and 700 μm. In three cases, BDA-positive neurons were located mainly within the boundaries of the area, whereas in the other 3 rats BDA spread also to neighboring portion of the lateral area (Figures 2B,C, 3E).

In the case of the *caudolateral part of the perifornical area*, the sizes of injections varied between 200 and 450 μm (Figures 2B,C, 3F). The 2 smaller injections centered within the area (Figure 2C), whereas the third, a large one, spread partly into the neighboring LHd (Figure 2B).

#### Lateral hypothalamic area

Five injections were centered in the lateral hypothalamic area: two injections in the ventral and three in the dorsal parts. (In this study, the term of “lateral hypothalamic area” is restricted to the posterior portion of the “hypothalamus-long” lateral hypothalamic area that extended between 2.4 and 3.4 mm caudal to the bregma level.) In the present study this area was arbitrary divided into dorsal and ventral areas by an imaginary line between the lower edge of the fornix and the centromedial point of the cross-sectional profile of the cerebral peduncle (Figures 1J–L, 2A–C).

Both injections into the *ventral part of the lateral hypothalamic area* were located entirely inside the boundaries of the area. Their sizes were moderate with average diameters around 400 μm (Figures 2C, 3H).

The sizes of injections into the *dorsal part of the lateral hypothalamic area* varied between 250 and 350 μm. In two rats, all of the BDA-labeled cells were within the boundaries of the area, whereas, in the other case, a small spread of label was seen in the LHv (Figures 2A,C, 3G).

### Distribution of BDA-positive nerve fibers in the lower brainstem

#### Midbrain

**Figure 4:**
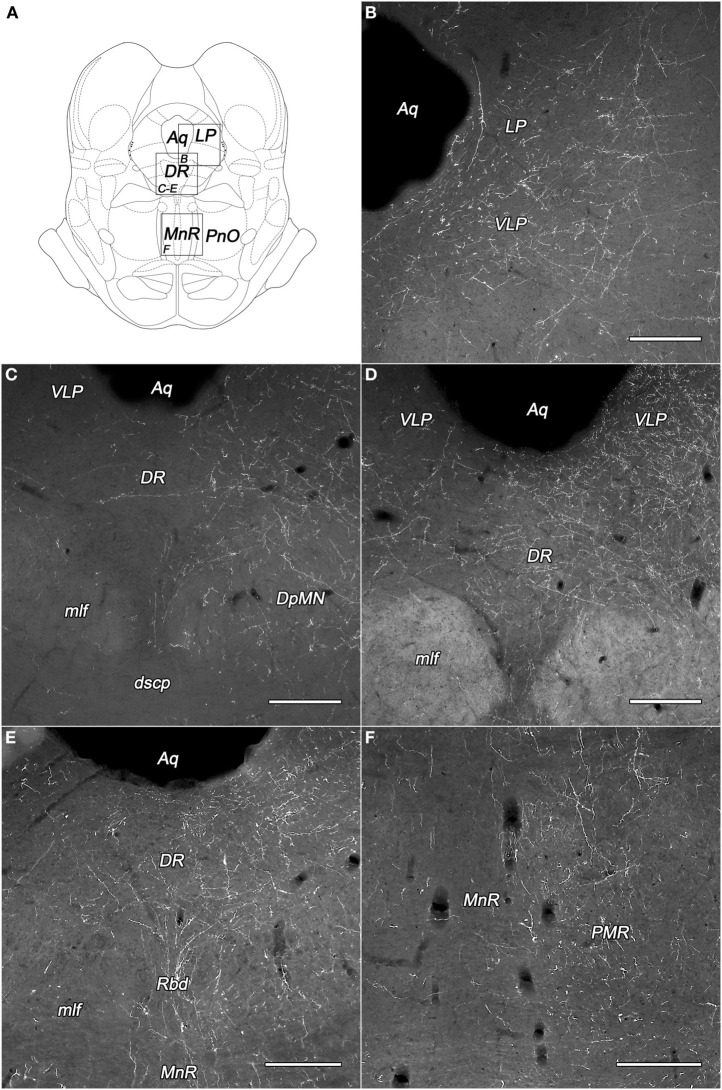
**Labeled fibers in the periaqueductal central gray (PAG) and the midbrain raphe nuclei following BDA injections into different subregions of the DLH. (A)** Drawings for outlining of the corresponding midbrain areas on panels B–F, according to the atlas of Paxinos and Watson (2007), with modifications. **(B)** Fine BDA-containing fiber network in the lateral and ventrolateral PAG originating from the rostral perifornical area. **(C–E)** Varicose BDA-containing fibers in dorsal raphe nucleus originating from the rostral **(C,D)** and caudolateral **(E)** perifornical area. **(F)** Moderately dense network of varicose fibers in the paramedian raphe nucleus after injections into the caudolateral part of the perifornical area. Notes: the unilateral BDA-injections were located on the right side. Ipsilateral dominance of labeled fibers can be seen on panels **(C–F)**. Aq, cerebral aqueduct; DpMN, deep mesencephalic nucleus; DR, dorsal raphe nucleus; dscp, decussation of the superior cerebellar peduncle; LP, lateral periaqueductal gray matter; mlf, medial longitudinal fascicle; MnR, median raphe nucleus; PMR, paramedian raphe nucleus; PnO, oral part of the pontine reticular nucleus; Rbd, rhabdoid nucleus; VLP, ventrolateral periaqueductal gray matter. Scale bars: 300 μm.

In general, the locations of BDA-positive fibers in the midbrain (Figure 4A) showed ipsilateral dominance to the unilateral application of the tracer in the DLH (Figures 4C–F).

The most densely network of labeled fibers in the midbrain appeared in the periaqueductal central gray (PAG). They arise mainly in the three subdivisions of the perifornical area and the periventricular subdivision of the dorsomedial hypothalamic area (Table 1). Dense network of BDA-positive fibers was seen in the ventrolateral and the ventral parts (Figures 4B,D), moderate to weak in the other parts of the PAG. Although, a substantial portion of the fibers appeared to be “axons of passage,” there were also interspersed numerous axons with varicosities, indicating possible synaptic contacts.

Numerous axons fanned out in the midbrain lateral and ventral to the PAG, predominantly in the deep mesencephalic (also called cuneiform) nucleus, the precuneiform area, and in the isthmic reticular formation (Figures 4C–E).

The dorsal raphe nucleus established moderate to heavy distribution patterns of BDA-positive fibers originated from the PeF and LHv subdivisions of the DLH (Figures 4C–E). In the median raphe nucleus (also known as midbrain raphe or superior central raphe nucleus) only light or very low projections were observed (Figure 4F). The rhabdoid and paramedian raphe nuclei showed moderate density of BDA fibers (Figures 4E,F, respectively).

The other investigated midbrain nuclei and areas, including the oculomotor and trochlear nuclei showed low fiber densities or were devoid of BDA-positive fibers (Table 1).

#### Pons

Like in the midbrain, the distribution of the BDA fibers was bilateral but with strong ipsilateral dominance.

A moderate density of partly *en passant* BDA-positive fibers was apparent within the oral part of the pontine reticular formation after injections into the PeFcm and PeFcl, and lighter densities after the other injections. Light to moderate density of labeling was observed in both the oral and caudal parts of the pontine reticular formation especially after injections into PeV, PeFr, and LHv (Table 1).

Labeled fibers were present in the pedunculopontine and the laterodorsal tegmental nuclei after all injections into the 8 subdivisions. Their density was very high after LHv and moderate after PeFcl injections (Table 1).

**Figure 5:**
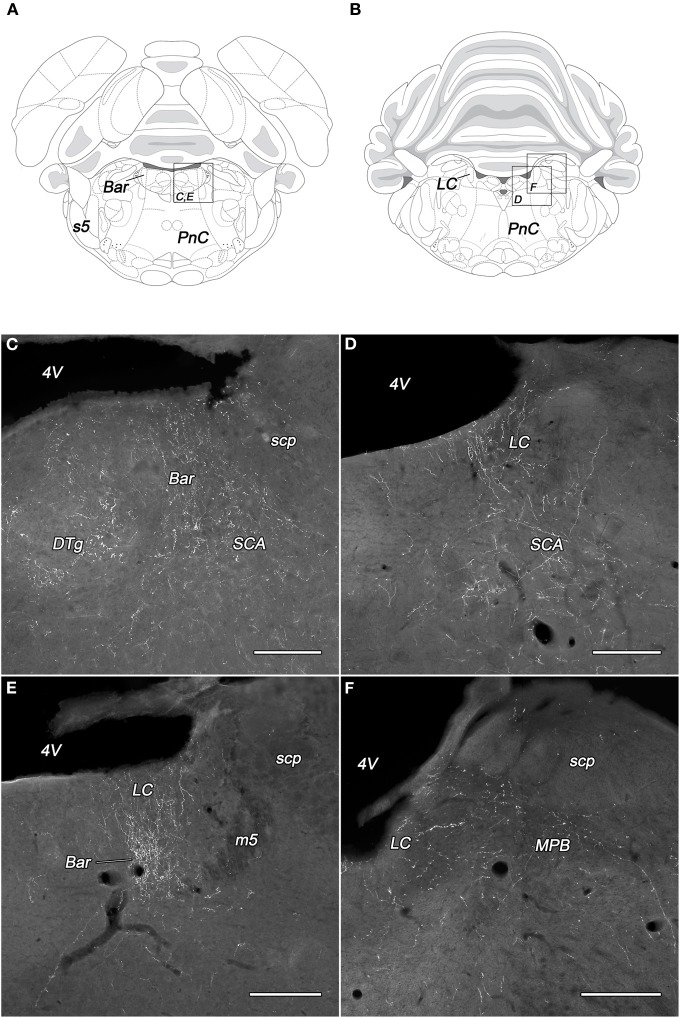
**Labeled fibers in the locus coeruleus and the Barrington's nucleus following BDA injections into different subregions of the DLH**. Drawings with outlined areas for panels C and E **(A)** and panels D and F **(B)** according to the atlas of Paxinos and Watson (2007), with modifications. Fine, moderately dense varicose fibers are seen in the Barrington's nucleus, the dorsal tegmental nucleus, and the subcoeruleus area following caudomedial perifornical injections **(C)**. BDA-containing fibers in the rostral locus coeruleus originating from the caudomedial perifornical area **(D)**. Densely labeled rostral locus coeruleus and Barrington's nucleus after periventricular injections **(E)**. Fine, varicose BDA fibers in the caudal locus coeruleus and medial parabrachial nucleus following BDA injection into the rostral perifornical area **(F)**. Bar, Barrington's nucleus; DTg, dorsal tegmental nucleus; LC, locus coeruleus; m5, mesencephalic trigeminal tract; MPB, medial parabrachial nucleus; PnC, caudal part of the pontine reticular nucleus; s5, sensory root of the trigeminal nerve; SCA, subcoeruleus area; scp, superior cerebellar peduncle; 4V, fourth ventricle. Scale bars: 300 μm.

Dense network of BDA-positive fibers was seen in the Barrington's nucleus but only in PeV injected rats (Figures 5A,C,E). The density of the BDA-labeled fibers in the locus coeruleus was almost equal to those in the Barrington's nucleus. The PeV and the subdivisions of the PeF generated moderate, while the other subdivisions of the DLH only light (or sparse in the case of DMH) projections to the locus coeruleus (Figures 5D,E). In the caudal portion of the locus coeruleus, from where neurons projects to the spinal cord, the labeling of fibers were rare (Figures 5B,F). Ventral to the locus coeruleus, in the subcoeruleus area, especially in its dorsal part, moderately dense fiber network was present. The fibers were seen mainly in rats with injections in the PeFr, the PeFcm, and the LHv (Figures 5C,D). BDA-positive fibers were present in very light to moderate densities in the lateral and medial parabrachial nuclei. The neurons of PeFr and PeFcl innervated moderately both parabrachial nuclei.

In the ventrolateral area of the pons, few fibers were spread throughout the area of the A5 noradrenaline cell group. Few labeled fibers were seen in the pontine raphe and pontine nuclei, some in the sensory and motor nuclei of the pons (Table 1), while in many others, like sensory trigeminal, cochlear, and lateral vestibular nuclei were devoid of BDA-labeled fibers or terminals (not shown).

#### Medulla oblongata

Although generally, sparse labeled fibers could be traced all along the medulla, their topographical distributions and density were heterogeneous. They showed generally bilateral appearance with ipsilateral dominance.

**Figure 6:**
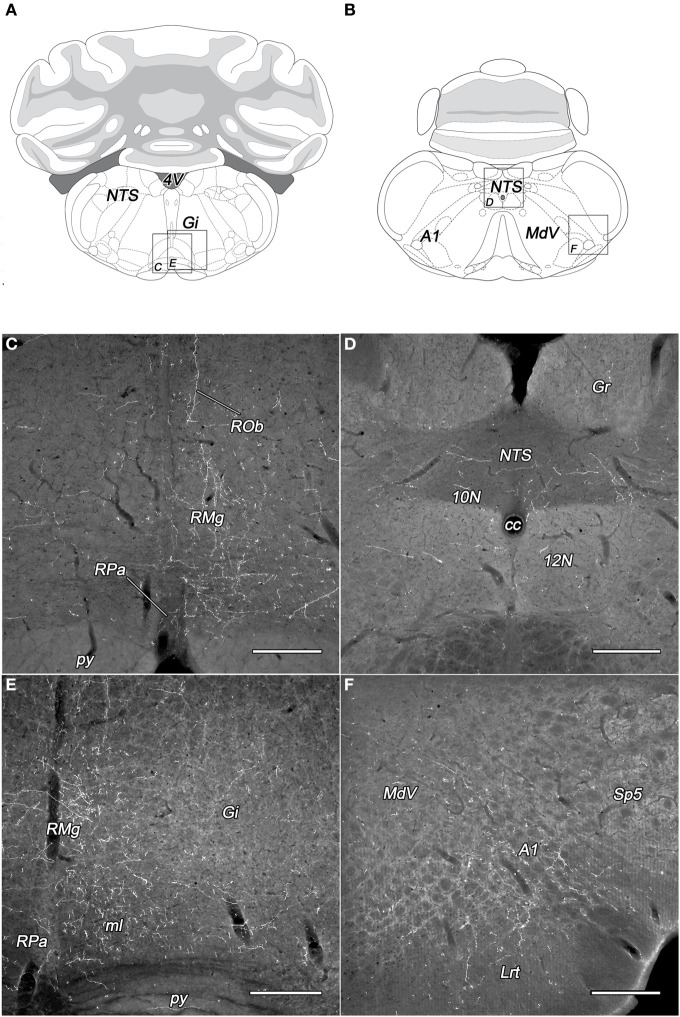
**Labeled fibers in the lower brainstem following BDA injections into different subregions of the DLH**. Drawings with outlined areas for panels C and E **(A)** and panels D and F **(B)** according to the atlas of Paxinos and Watson (2007), with modifications. Dense networks of BDA-containing fibers in various densities were seen in the raphe pallidus, obscurus, and magnus nuclei originating from the caudomedial perifornical area **(C,E)**. The distribution of the BDA-containing fibers is mainly ipsilateral to the injections **(C)**. Varicose BDA-labeled fibers were found in the caudal part of the nucleus of the solitary tract **(D)**, and in the A1 catecholamine cell group **(F)** following BDA injection into the caudolateral perifornical area. A1, A1 noradrenaline cell group; cc, central canal; Gi, gigantocellular reticular nucleus; Gr, gracile nucleus; Lrt, lateral reticular nucleus; MdV, ventral part of the medullary reticular nucleus; ml, medial lemniscus; NTS, nucleus of the solitary tract; py, pyramidal tract; RMg, raphe magnus; ROb, raphe obscurus; RPa, raphe pallidus; Sp5, spinal trigeminal nucleus; 10N, dorsal motor nucleus of vagus; 12N, hypoglossal nucleus; 4V, fourth ventricle. Scale bars: 300 μm.

The subdivisions of the DLH generated light labeling in the gigantocellular reticular nucleus (Figures 6A,C,E). In the vicinity, somewhat lesser density of fibers was observed in the lateral paragigantocellular and the intermediate reticular nucleus. Except neurons in the DMN, labeled fibers arise in the other seven subdivisions of the DLH, but in different densities (Table 1).

From all of the DLH divisions (with exception of DMN) projections were generated to the nucleus of the solitary tract: light ones from neurons of the PeV, PeFr, and LHv, and sparse projections from the other areas (Figures 6B,D). It was not the case for the dorsal motor nucleus of the vagus, where the BDA-labeling was low or sparse.

In the territory of the medullary catecholamine cell groups, despite of the gap of heavy innervations, the BDA fibers originated from more individual subdivisions of the DLH (Table 1). The source of the fibers was especially wide in the case of A2 noradrenaline cell group, which is innervated by *all of the investigated* DLH subdivisions. BDA-labeled fibers in the caudal part of the A1 noradrenaline (Figures 6B,F) and C1 adrenaline cell groups arised in 7 subdivisions, whereas the rostral part of the A1 noradrenaline and the C2 adrenaline cell groups received fibers from 5 different subregions of the DLH.

Light to moderate density of varicose fibers was present in the raphe magnus nucleus originating from all the subdivisions of the DLH (Figures 6C,E, Table 1). In the raphe pallidus and obscurus nuclei delicate varicose fibers were apparent after the tracer injections within 7 different subregions of the DLH (Figures 6C,E, Table 1). Some of the BDA-positive fibers traveled in the raphe obscurus and the paramedian reticular nucleus nearly perpendicular to the plane of the sections (Figure 6C).

In most cases, after perifornical or lateral hypothalamic injections, BDA-positive fibers were identified among fibers of large neuronal tracts or fasciculi. Their number was low, except in the medial lemniscus, the medial longitudinal fascicle, and the superior cerebellar peduncle, where the density of BDA fibers could be rated as moderate (Table 1).

Several, more than one third of recognized lower brainstem areas/nuclei/subdivisions (Paxinos and Watson, 2007) were devoid of BDA-labeled fibers or terminals. They are not listed on Table 1.

## Discussion

### Topography of the areas and subdivisions of the dorsolateral hypothalamic area

The DLH can be divided into three areas (Murphy et al., 2003; Harris et al., 2005) and further divided into 8 subdivisions (Figures 1A–L).

#### Dorsomedial hypothalamic area

This area consisted of 3 subdivisions. Each of them have been delineated and showed in one of the most popular topographical maps in the rat brain (Paxinos and Watson, 2007).

The *posterior periventricular area* is labeled as “Pe” (periventricular nucleus), expands from 2.24 to 3.48 mm caudal to the level of the bregma. This area is about 200–300 μm wide along the posterior portion of the third ventricle and arching over it (Figures 1D–F). This portion is called as “PVi” (intermediate hypothalamic periventricular nucleus) by Swanson (1987, 2004) and Hahn and Swanson (2010). The caudal part of the posterior periventricular area (between 2.92 and 3.48 mm) is also called as dorsal part of the posterior hypothalamic area (PHD) by Paxinos and Watson (2007). This is an area with relatively low neural cell density (Palkovits, 1975), cells are comprised of layers between the third ventricle and the dorsomedial nucleus.

The *hypothalamic dorsomedial nucleus* (Figures 1D–F) is made up of three subdivisions (dorsal, compact, and ventral). The medial and the dorsolateral portions of the dorsal division can be separated by their cellular density (Palkovits, 1975). The medial one is densely packed with neuronal cells, in contrast to the sparsely populated dorsolateral territory. This subdivision (designated as DMD in the atlas of Paxinos and Watson, 2007) extends rostro-caudally between 2.28 and 3.48 mm from the bregma level. Dorsolaterally, it is bordered by the dorsal hypothalamic area (see next).

The *dorsomedial hypothalamic subdivision* (signed also as dorsal hypothalamic area and labeled DMA by Bernardis and Bellinger, 1993 and DA by Paxinos and Watson, 2007) is comprised by various cell types with low cellular density. It can be separated from the zona incerta (dorsally), dorsomedial nucleus (ventromedially), the perifornical nucleus (ventrolaterally), and partly from the lateral hypothalamic area, laterally (Bernardis and Bellinger, 1993). It extends between 2.48 and 3.24 mm, rostrocaudally, from it is replaced by the most cellular dense posterior hypothalamic nucleus (Paxinos and Watson, 2007).

#### Perifornical area

Originally, this area has been described as perifornical nucleus (Gurdjian, 1927; Christ, 1969; Bleier et al., 1979). Various cell types with high density are located over the fornix and in small areas around the fornix 2.64–3.60 mm caudal to the bregma (Paxinos and Watson, 2007). Actually, the perifornical *area* is larger than the perifornical *nucleus*, and extends all directions, occupying the less cell dense territories dorsomedially and dorsolaterally to the fornix. Correctly, the perifornical nucleus (signed PFn Palkovits, 1975, PFX by Geeraedts et al., 1990, PeF by Paxinos and Watson, 2007) consists only a part of the perifornical area (that is labeled PeFLH by Paxinos and Watson, 2007). The “nucleus” and the “area” can be clearly differentiated by their cellular density and somewhat by their cell types (Palkovits, 1975). The perifornical area extends 2.16–3.48 mm caudally to the bregma level (Paxinos and Watson, 2007). Dorsally, the perifornical area fuses with the medial part of the zona incerta. Medially, a relatively loose area separate the perifornical area from the dorsomedial nucleus and dorsomedial hypothalamic subdivision, like laterally from the lateral hypothalamic area (Palkovits, 1975). The perifornical area was divided rostral and caudal portions by Geeraedts et al. (1990).

The *rostral perifornical subdivision* (2.4 and 2.8 mm caudal to the level of the bregma; Figure 1G) contains relatively large cells (Palkovits, 1975) and higher cellular density. This subdivisions extends not only dorsally, but somewhat medial to the fornix (Paxinos and Watson, 2007).

The caudal part of the perifornical nucleus and area is larger and longer (2.8 and 3.4 mm caudal to the bregma) than the rostral one. It extends more dorsally, up to nearly the mamillothalamic tract. We divided the caudal part into medial and lateral parts by an imaginary line through the fornix (Figures 1H,I). The cellular density in the *caudomedial perifornical subdivision* is higher than in the *caudolateral subdivision*. Besides this, the caudomedial subdivision establishes more heterogeneous cell population (Palkovits, 1975).

#### Lateral hypothalamic area

This area constitutes a segment of the lateral hypothalamic area between 2.4 and 3.6 mm caudal to the bregma level. It is also called “peduncular part” of the lateral hypothalamus (PLH) by Paxinos and Watson (2007). Laterally, it extends to the subthalamic area, where it is bordered by the cerebral peduncle/internal capsule and the optic tract. It is bordered by the perifornical area, dorsomedially, and by the ventromedial nucleus, ventromedially. Dorsally, it is covered by the zona incerta and the Forel field (Paxinos and Watson, 2007). The area is used to be divided into a higher cell-dense *ventral* and a lesser cell-dense *dorsal* subdivisions of the lateral hypothalamic area (Geeraedts et al., 1990; Bernardis and Bellinger, 1993; Swanson, 2004; Paxinos and Watson, 2007). However, the actual borderline between these two areas is poorly defined. Two versions have been reported, or indicated in maps: (1) divided the ventral and dorsal parts by an imaginary line between the fornix and the midpoint of the medial borderline of the cerebral peduncle (Bernardis and Bellinger, 1993; Swanson, 2004). This is an oblique line moving upward medial to lateral direction; (2) imaginary horizontal line from the fornix, lateralwards (Geeraedts et al., 1990). We applied the first version in our study (Figures 1J–L).

### Methodological considerations

1. Although, the obligatory factors to be verified, like the tip diameter of the capillary, current, duration of the injections, post-injection time, the histotechnical procedure were standardized, there were differences in the size of injections. In addition of above factors, the variability of the histological structures in the target areas, such as the cellular density or density of local fiber network, and the capillary density may also cause some differences in the final size of injection areas.
2. Similar to the injection sites, the size of the nucleus or area creates evaluation problems. In general, the total area of any particular nuclei or subdivisions does not filled up completely (100%) during tract-tracing studies, especially not the entire (three-dimensional) volume of these structures. In spite of this fact, during evaluation of fillings or rating densities, nuclei or subdivisions are usually counted or quantified as equal units. In both cases, the comparison of individual data needs caution and appropriate criticism.
3. During subjective evaluations, we used categories for the fiber density (like high, moderate, low, scattered), like it is frequently applied in tract-tracing studies, or descriptive neuroanatomy. In such case, the subjective evaluations could be acceptable for comparison data among groups rather than comparison individuals. The correct delineation of the innervation areas, appropriately detailed information for experimental parameters, enough sections from a good number of animals are basic requirements. We tried to fulfill all these criteria.

### Distribution patterns of DLH projections on the lower brainstem

Although, the present findings confirm a significant portion of the data reported about the innervation pattern of the dorsolateral hypothalamus (DLH) to the lower brainstem *in general*, the evaluation of the projections of the *8 individual subgroups* provides us new information.

1. *The lower brainstem is widely innervated by neurons in the DLH*. In general, projections of the DLH to the lower brainstem is rather low than moderate, but the number of the innervated areas/nuclei are relatively high. Among of the 194 reported nuclei, subdivisions and areas (Paxinos and Watson, 2007) 116 contained BDA fibers, in various density.
  Within the lower brainstem two, sometimes three descending pathways (dorsal, ventromedial, ventrolateral) have been described (Roeling et al., 1994; Peyron et al., 1998; Goto et al., 2005; Hahn and Swanson, 2010, 2012). Reportedly, a substantial percentage of the fibers in these pathways arise in neurons of the DLH, especially in the perifornical area (Roeling et al., 1994). We could not observe any BDA-labeled fibers after injections into any of the investigated subdivisions that formed definitive bundles or fasciculi. It is noteworthy, that BDA-labeled fibers join in different extent to shorter or longer portions of 13 known tracts/fasciculi (Table 1). They descended rather like solitaire fibers parallel to the rostro-caudal axis of the lower brainstem. It seems to be most probably, that the injections into the individual subdivisions of the area were very small compare to those infiltrated larger portions of the DLH. Consequently, the possible aggregation of the parallel-running labeled fibers was minimal, or at least minor to provide us an impression of fiber bundles or fascicles.
2. *The participation of DLH areas and subdivisions in the innervations of the lower brainstem is not equal*. Based on observations from serial sections of brainstems from 22 rats, we concluded that the projection patterns in the lower brainstem of the 8 subdivisions are distinct. From the 3 subdivisions of the perifornical area twice as many brainstem areas/nuclei (90–99) received fibers than from the 3 subdivisions of the dorsomedial hypothalamic area (26–58). From the lateral hypothalamic area, neurons from the ventral part projected to more brainstem areas (83) than neurons from the dorsal one (69). Only 13 nuclei/areas received BDA-labeled fiber from all of the 8, and additional 25 from 7 subdivisions.
  The most dense projections from the investigated 8 subdivisions of the DLH arisen partly from the three perifornical ones. The widest range of lower brainstem areas with labeled fibers were found after perifornical injections (Table 1). The distribution patterns of fibers of perifornical origin are similar to those published Hahn and Swanson (2010), with minor differences. The differences were not seen in the topographical distribution patterns rather than in the density of labeled fibers in certain brain areas. These differences could be derived from the method of evaluation, or from the volume of tracers injected into the perifornical areas *vs*. subdivisions.
  Moderate to low labeling were seen in the lower brainstem following tracer injections into the ventral part of the lateral hypothalamic area. The distribution patterns of labeled fibers were somewhat different, but the intensity of labeling, in general was the same. Injections into the 3 dorsomedial subdivisions resulted in a variation of the density of labeled fibers, especially after periventricular applications.
3. *Few lower brainstem areas/nuclei are highly innervated by some or more DLH subdivisions*. Among the 116 nuclei/areas where BDA-positivity was observed, the heavy innervation patterns represented only in four: in the lateral and ventrolateral parts of the periaqueductal gray, in the Barrington's nucleus after periventricular, and in the pedunculopontine tegmental nucleus after ventral lateral hypothalamic injections (Table 1).
  The observation about the highly innervated ventral and ventrolateral parts of the PAG is supported by several previous descriptions (Goto et al., 2005; Hahn and Swanson, 2010, 2012). The caudal part of the PAG contains fibers from the hypothalamic dorsomedial nucleus (Thompson et al., 1996). In the present study, some labeled fibers were observed after BDA-injection into the DMN, which did not represent the entire dorsomedial nucleus in our case, only the lateral portion of its dorsal part (see Introduction). The dorsomedial hypothalamic area incorporates a part of the A13 dopamine cell group. Recently, it has been demonstrated that dopamine-containing fibers from the A13 cell group project to the dorsolateral PAG (Messanvi et al., 2013).
  The BDA-labeled fibers extended further caudal to the pontine tegmentum, especially to the Barrington's nucleus, where very dense labeling was found following periventricular injection. Hahn and Swanson (2010) also reported intense labeling but following perifornical tracer application. Our data partly confirm data published by Thompson et al. (1996) and Allen and Cechetto (1992) reporting fibers in the Barrington's nucleus, but far less than after tracer injections into the periventricular area. Moderate dense fiber networks were seen in the laterodorsal tegmental and the parabrachial nuclei (Table 1). These observations are in agreement with earlier studies (Goto et al., 2005; Hahn and Swanson, 2010, 2012).
4. *Some information about the distinct innervations of the different parts of the reticular formation*. From the subdivisions of the DLH 20 nuclei/parts of the lower brainstem reticular formation receive fibers. The innervation occurence, not considering the dorsomedial nucleus, is high (70–90%), but the density is generally low. It should be mentioned that due to the structure of the reticular formation, a certain percentage of the BDA fibers could be fragments of “axons of passage.”
5. *Participation of DLH areas and subdivisions in the innervations of biogenic amine cell groups in the lower brainstem*. The presence of BDA-positive fibers was individually examined in nine-nine noradrenaline and serotonin, and additional 3 adrenaline cell groups. The vast majority of the fibers arisen from the 3 perifornical and the two lateral hypothalamic subdivisions. Each of the cell groups received BDA fibers, most of them at least from 6 subdivisions. The density of labeled fibers varied from scattered to moderate. From the 25 lower brainstem nuclei/areas, which were moderately innervated by one of the DLH subdivisions, 18 belonged to the monoamine cell groups.
  The lower brainstem catecholamine neurons are significant participants of the *ascending reticular activating system* running from the lower brainstem to forebrain areas. Descending fibers from the DLH to the participants of this system may be carriers of functionally important feedback signals.
  In each case, labeled fibers appeared to cross the midline just caudal to the sites of injections. The potential crossover routes include the PAG at the dorsal raphe nucleus, the pontine tegmentum, and the territory of the nucleus of the solitary tract in the medulla.
  Two distinct neuropeptides, orexin- and melanin-concentrating hormone (MCH) have been localized in the DLH (Skofitsch et al., 1985; Bittencourt et al., 1992; Peyron et al., 1998; Nambu et al., 1999; Hahn, 2010; Bittencourt, 2011). Both of them are important components of the central regulation of food intake and energy balance, as well as the sleep-wake cycle (Bittencourt, 2011; Barson et al., 2013; Inutsuka and Yamanaka, 2013). From here, both orexin- and MCH-containing nerve fibers distribute throughout the entire central nervous system, and send innervations to numerous brainstem areas and nuclei (Skofitsch et al., 1985; Bittencourt et al., 1992; Peyron et al., 1998; Nambu et al., 1999; Bittencourt, 2011). The topographical distribution of these two neuropeptides in the lower brainstem has been mapped in details (Bittencourt et al., 1992; Peyron et al., 1998). Their distribution patterns show significant differences from each other (Bittencourt et al., 1992; Peyron et al., 1998). Their combined distribution pattern shows a fairly large overlap with the distribution of BDA-labeled fibers in the present study, but this overlap is not complete. It seems to be obvious, that the labeled fibers may be arisen in either orexin- or MCH-expressing neurons in the DLH, or from some other neuropeptide- or neurotransmitter-containing sources, even in a small percentage.
  The moderate density of BDA-labeled fibers in the catecholamine- and serotonin-expressing lower brainstem areas overlap with the orexin or MCH innervations of these areas. Orexin- and MCH-containing axon terminals were found in the locus coeruleus (Horvath et al., 1999; Yoon and Lee, 2012), their fibers distribute in A1, A2 noradrenaline, and C1 and C2 adrenaline cell groups with orexin synaptic contacts (Puskas et al., 2010). Practically, all of these aminergic cell groups established BDA-positive fibers following injections to all of the DLH subdivisions.
  The presence of orexin- and MCH-containing axon terminals in the dorsal raphe nucleus was reported (Wang et al., 2003; Yoon and Lee, 2012). A large density of orexin- and MCH-positive fibers was demonstrated in the median raphe (Peyron et al., 1998; Hill et al., 2013), the raphe pallidus and obscurus (Berthoud et al., 2005; Tupone et al., 2011). Labeled fibers in moderate to low densities were seen in all of these raphe nuclei after BDA injections into the subdivisions of the DLH (Table 1).
  The wide distribution of DLH fibers in the lower brainstem may provide anatomical basis for the multiple functional activity of neurons located in the DLH, especially those express orexin and MCH.

## Conclusion

The main goal of the present study was to localize the origin of lower brainstem projections of individual subdivisions of the dorsolateral hypothalamus (DLH). The topographical distribution of labeled fibers has been localized in the lower brainstem following applications of an anterograde tracer into 8 distinct subdivisions of the dorsolateral hypothalamic area. The observations obtained from mosaic-like analysis of projections from the 8 subdivisions of the DLH may provide us relatively complete information about the lower brainstem projections of the DLH. The 60% of the brainstem structures (nuclei, areas, subdivisions) receive input from the dorsolateral hypothalamus. The projection patterns of the 8 investigated subdivisions of the DLH to the lower brainstem are all individual. There is limited number of brainstem areas with high label density (only 4 of 116 investigated areas), but there are specific cell groups (noradrenaline and serotonin cell groups, several nuclei/areas of the reticular formation) where the density of the fibers of DLH origin is higher than the average in the lower brainstem. Since lower brainstem includes important autonomic and visceral regulatory areas and nuclei, the reticular formation, as well as the central noradrenaline and adrenaline neurons (including the premotor sympathetic ones), serotonin-expressing raphe nuclei, the knowledge of the exact sites of their neuronal inputs are of functional significance.

## Author contributions

The first author contributed to the stereotaxic surgery and tissue preparation. Both authors contributed to the analysis of results, the preparation of the figures and table, and the writing of the manuscript.

### Conflict of interest statement

The authors declare that the research was conducted in the absence of any commercial or financial relationships that could be construed as a potential conflict of interest.

## Abbreviations

A1: A1 noradrenaline cell group
ABC: avidin-biotin-horseradish peroxidase complex
Aq: cerebral aqueduct
Bar: Barrington's nucleus
BDA: biotinylated dextran amine
cc: central canal
DLH: dorsolateral hypothalamic area
DM: dorsomedial hypothalamic area
DMH: dorsomedial hypothalamus
DMN: dorsomedial nucleus
DR: dorsal raphe nucleus
dscp: decussation of the superior cerebellar peduncle
DTg: dorsal tegmental nucleus
DV: dorsoventral coordinates
F: fornix
Gi: gigantocellular reticular nucleus
Gr: gracile nucleus
L: lateral coordinates
LC: locus coeruleus
LH: lateral hypothalamic area
LHd: dorsal part of the lateral hypothalamus
LHv: ventral part of the lateral hypothalamus
Lrt: lateral reticular nucleus
MCH: melanin-concentrating hormone
MdV: ventral part of the medullary reticular nucleus
mlf: medial longitudinal fascicle
MnR: median raphe nucleus
MT: mamillothalamic tract
Ni-DAB: nickel enhanced 3,3-diaminobenzidine
NTS: nucleus of the solitary tract
OT: optic tract
PAG: periaqueductal central gray
PeF: perifornical area
PeFcl: caudolateral perifornical area
PeFcm: caudomedial perifornical area
PeFr: rostral perifornical area
PeV: periventricular area
PMR: paramedian raphe nucleus
PnC: caudal part of the pontine reticular nucleus
PnO: oral part of the pontine reticular nucleus
py: pyramidal tract
Rbd: rhabdoid nucleus
RC: rostrocaudal coordinates
RMg: raphe magnus
ROb: raphe obscurus
RPa: raphe pallidus
s5: sensory root of the trigeminal nerve
SCA: subcoeruleus area
Sp5: spinal trigeminal nucleus
VLP: ventrolateral periaqueductal gray matter
10N: dorsal motor nucleus of vagus
12N: hypoglossal nucleus
3V: third ventricle
4V: fourth ventricle.
